# Dissecting Intra-Tumor Heterogeneity by the Analysis of Copy Number Variations in Single Cells: The Neuroblastoma Case Study

**DOI:** 10.3390/ijms20040893

**Published:** 2019-02-19

**Authors:** Federica Cariati, Francesca Borrillo, Varun Shankar, Marcella Nunziato, Valeria D’Argenio, Rossella Tomaiuolo

**Affiliations:** 1 Dipartimento di Medicina Molecolare e Biotecnologie Mediche, Università di Napoli Federico II, Via Sergio Pansini 5, 80131 Naples, Italy; cariati@ceinge.unina.it (F.C.); francescaborrillo@gmail.com (F.B.); cv11081993@gmail.com (V.S.); nunziato@ceinge.unina.it (M.N.); 2 CEINGE-Biotecnologie Avanzate, Via Gaetano Salvatore 486, 80145 Naples, Italy; 3 KronosDNA srl, Spin-Off of Università di Napoli Federico II, Via Loggia dei Piasani 25, 80133 Naples, Italy

**Keywords:** intra-tumor heterogeneity, single cell isolation, whole genome amplification, next-generation sequencing, copy number variations, chromosomal pattern

## Abstract

Tumors often show intra-tumor heterogeneity because of genotypic differences between all the cells that compose it and that derive from it. Recent studies have shown significant aspects of neuroblastoma heterogeneity that may affect the diagnostic-therapeutic strategy. Therefore, we developed a laboratory protocol, based on the combination of the advanced dielectrophoresis-based array technology and next-generation sequencing to identify and sort single cells individually and carry out their copy number variants analysis. The aim was to evaluate the cellular heterogeneity, avoiding overestimation or underestimation errors, due to a bulk analysis of the sample. We tested the above-mentioned protocol on two neuroblastoma cell lines, SK-N-BE(2)-C and IMR-32. The presence of several gain or loss chromosomal regions, in both cell lines, shows a high heterogeneity of the copy number variants status of the single tumor cells, even if they belong to an immortalized cell line. This finding confirms that each cell can potentially accumulate different alterations that can modulate its behavior. The laboratory protocol proposed herein provides a tool able to identify prevalent behaviors, and at the same time highlights the presence of particular clusters that deviate from them. Finally, it could be applicable to many other types of cancer.

## 1. Introduction

It is widely accepted that cancer is a highly heterogeneous disease and that subpopulations of cells, within a single tumor, can exhibit distinct genomic profiles. Indeed, the ensemble behaviors of a cellular population may not represent the behavior of any individual cell [1]. Recent technological advances made it possible to analyze nucleic acids and proteins from different areas of a single tumor, as well as within a heterogeneous tumor sample, reaching single-cell resolution. In this way, it is possible to avoid the averaging of bulk analysis and to capture the heterogeneity of cells [2].

In the case of Neuroblastoma (NB), where high cellular heterogeneity is a hallmark, the wide range of clinical presentations and the uneven response to treatment seem to be due to cellular heterogeneity [3]. NB heterogeneity is related to tumor differentiation and histology: it derives from multipotent Neural Crest Cells (NCCs), forms during embryonic development, and mainly involves the sympathetic nervous system (abdomen, especially the adrenal gland) [4,5]. 

The occurrence of NB is at early infancy and childhood, almost all NB cases being diagnosed at the age of 10 (98%). NB survival rate depends on the success of the treatment and the rate of diagnosis [6]. Nearly 50% of patients have a localized tumor (primary site) with an average survival rate of about five years, while in the other 50% of patients, it occurs in an advanced, final stage [7]. Generally, patients aged more than 1.5 years at diagnosis have much worse outcomes than infants, and the presence of the metastatic stage denotes a poor prognosis [8]. Genetics plays a crucial role in the NB tumorigenesis; in fact, two key factors to identify the progression and prognosis of the tumor are the detection of structural Copy Number Variations (CNVs) and the amplification of the *MYCN* gene, able to identify those tumors with poor prognosis and rapid progression, independently of age and clinical stage [7,8,9]. However, *MYCN* amplification can only be seen in about 25% of NB patients; thus, other contributing factors that are still unknown or not tested have to be implicated in the other cases [10].

Sometimes, genetic variations, which affect only a small number of cells, can be undetectable, especially if the molecular analysis is performed on a larger mixed pool of normal and variant tumor cells [11]. As a consequence, the signal of the tumor cells that are driving the progression of the tumor could be hidden. The characterization of single cells would allow highlighting the presence of possible subpopulations or providing further information on the genetic identity of the cells.

Therefore, the purpose of this study was to develop a laboratory protocol that allows the evaluation of the cellular heterogeneity, avoiding incurring over- or under-estimation errors. We used a combination between the advanced DEPArray™ technology and Next-Generation Sequencing (NGS) to identify, manipulate, and sort single cells individually and then to carry out their CNV analysis. The presence of chromosomal alterations, some common to all cells and others specific to a few cells, first allowed identifying the cellular subpopulations and, subsequently, checking for genes that were located in those regions.

## 2. Results

The combined use of the DEPArray^TM^ technology platform with NGS allowed analyzing 33 single cells isolated from two neuroblastoma cell lines, namely SK-N-BE (2)-C and IMR-32.

**Figure 1:**
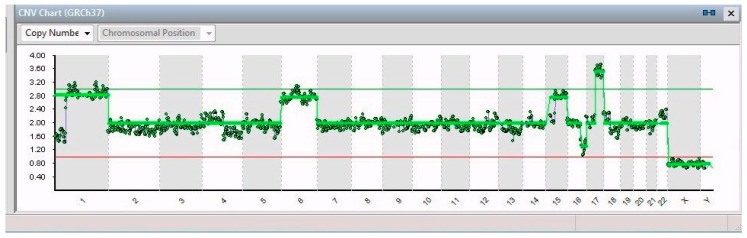
CNV chart related to a single cell from IMR-32 showing, from left to right, partial gain of chromosome 1, total gain of chromosomes 6 and 15, a partial loss of chromosome 16, a partial gain in chromosome 17, and the total loss of the X chromosome.

**Figure 2:**
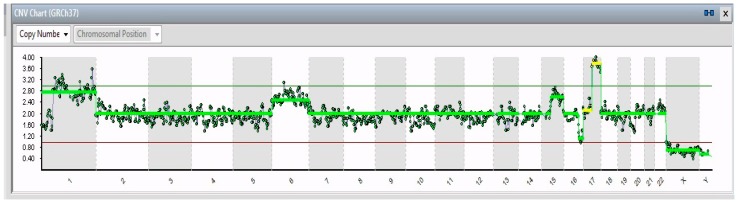
CNV chart related to a single cell from IMR-32 showing, from left to right, partial gain of chromosome 1, total gain of chromosome 6, partial gain of chromosome 15, a partial loss of chromosome 16, a partial gain of chromosome 17, and the total loss of the X chromosome.

Of the 24 cells isolated from the IMR-32 plate, 19 were considered suitable for the analysis of the chromosomal pattern, which allowed highlighting in all 19 IMR-32 single cells the presence of a total gain of chromosome 6, 2 partial gains, 1 in the chromosomal region between 1p32.3 and 1q44 (194 Mb) and the other in the chromosomal region between 17q21.31 and 17q25.3 (39 Mb), and a partial loss of the chromosomal region between 16q22.2 and 16q24.3 (18 Mb). Moreover, all cells showed a gain in chromosome 15, although it was total only in 15/19 cells (Figure 1) and partial (15q15.3–15q26.3) in the other 4 (Figure 2). Notable identifications were the total loss of chromosomes X (2/19) and 13 (1/19) and a partial loss of chromosome 11, i.e., 11p15.2–11p21 (42 Mb), 11q14.1–11q23.2 (32 Mb), ad 11q23.2–11q26.3 (21 Mb) in 1 cell.

All 14 isolated single cells from SK-N-BE (2)-C presented a partial gain of chromosomes 7 (7q32.1–q36.3 of 27 Mb) and 11 (11q13.3–11q25 of 65 Mb), a total loss of X chromosome, and a partial loss of chromosomes 3 (3p26.3–3p14.2 of 61 Mb), 13 (13q12.11–13q31 of 66 Mb), 17 (17p13.3–17q11.2 of 30 Mb), 19 (19q12–19q13.43 of 28 Mb) and 21 (21q22.2–q22.3 of 6 Mb).

**Figure 3:**
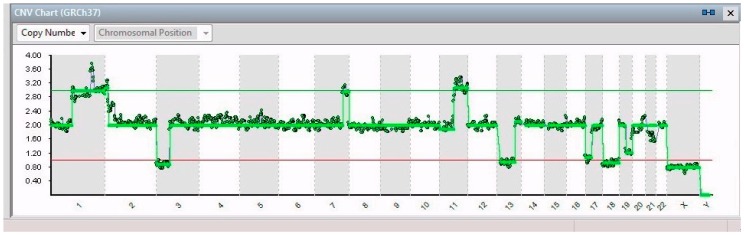
CNV chart related to a single cell from SK-N-BE (2)-C showing, from left to right, partial gain of chromosome 1, partial loss of chromosomes 3, partial gain of chromosomes 7 and 11, a partial loss of chromosomes 13, 17, 19, and 21, and the total loss of the X chromosome.

**Figure 4:**
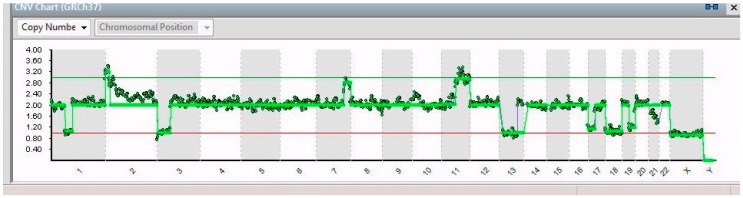
CNV chart related to a single cell from SK-N-BE (2)-C showing, from left to right, partial loss of chromosome 1, partial gain of chromosome 2, partial loss of chromosomes 3, partial gain of chromosomes 7 and 11, a partial loss of chromosomes 13, 17, 19, and 21, and the total loss of the X chromosome.

In 8/14 cells, a partial gain of chromosome 1 was found (1p32.3–1q44 of 151 Mb) (Figure 3); moreover, 5/14 cells showed a partial loss in that chromosome (1p32.2–1p21.3 of 44 Mb) (Figure 4); 6/14 cells had partial gain of chromosomal region between 2p25.3 and 2p21 (44 Mb); and just 1 cell showed a peculiar gain of chromosome 9, i.e., 9p24.3–9p23 (13 Mb).

**Figure 5:**
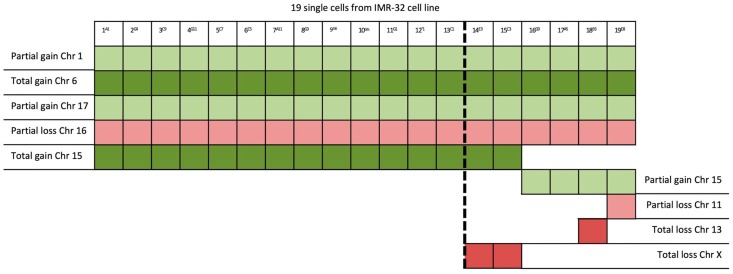
CNV evaluation of all 19 isolated single cells from IMR-32 revealed the existence of one main subpopulation (to the left of the dotted line) and 5 different chromosomal patterns: (I) from Sample 1–sample 13; (II) Samples 14 and 15; (III) Samples 16 and 17; (IV) Sample 18; (V) Sample 19.

**Figure 6:**
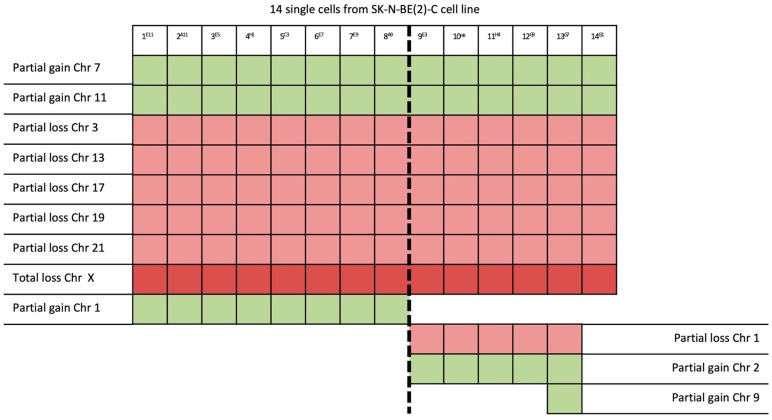
CNV evaluation of all 14 isolated single cells from SK-N-BE (2)-C revealed the existence of two main subpopulations within the same cell line and four different chromosomal patterns: (I) from Sample 1–Sample 8; (II) Samples 9 and 12; (III) Sample 13; (IV) sample 14.

Definitely, these results show that, among the 19 single cells isolated from the same IMR-32 cell line, 5 different chromosomal patterns were identified (Figure 5), and among the 14 single cells isolated from the SK-N-BE (2)-C cell line, 4 different chromosomal patterns were identified (Figure 6), highlighting the importance of the analysis at the single-cell level.

## 3. Discussion

Cancers often exhibit intra-tumor heterogeneity due to genotypic differences between individual cells present in the tumor itself. Molecular characterization of single cells is pivotal for a reliable genomic analysis, since it allows avoiding the loss of sensitivity derived from the analysis of samples in which different cells coexist or that derive from more cell clones.

The evaluation of NB cell heterogeneity had been previously approached; however, the chromosomal pattern at the single-cell level had never been tested before [3].

The protocol we have developed herein, thanks to both the analysis of the CNVs and the definition of chromosomal patterns, underlines the importance of the analysis at the single-cell level; indeed, for both SK-N-BE (2)-C and IMR-32, it was able to detect the presence of different chromosomal patterns within the same cell line. In addition, starting from the evaluation of chromosomal patterns, we first checked the presence of possible cell subpopulations (Figure 5 and Figure 6) and then looked for the genes present in the affected genomic areas by consultation of the gene bank software (Table 1 and Table 2).

Although the relationship between the presence of gain or loss of chromosomal regions and cancer has not been permanently established, it is certainly evident that the loss of tumor suppressor genes (chromosomal deletion) and the overexpression of oncogenes (chromosomal duplication) are consistent with the nature of cancer. For example, the amplified copies of oncogene *MYCN* located in the 2p25–p22 region confer resistance to some treatments used for NB therapy. Patients with amplified *MYCN* have markedly poorer prognosis than those in which *MYCN* copy number is not elevated [9].

Although the aim of this work is not to draw conclusions about the impact that the combination of more or less expressed genes may have on tumor progression, in Table 1 and Table 2, we report the main genes present in the affected genomic regions and the corresponding literature highlighting their implications for cancer.

In particular, concerning the IMR-32 cell line, the main subpopulation is characterized by the duplication of chromosomes 6 and 15, partial gain of chromosomes 1 and 17, and partial loss of chromosome 16 (Table 1). The presence of a supernumerary 6 chromosome in all the IMR-32 single cells analyzed can be related to the Single-Nucleotide Polymorphisms (SNPs), FLJ22536 and FLJ44180, in position 6p22, previously described to be associated with the sporadic form of NB [12]. In this same position, three SNPs, namely CASC15, CASC15-S, and CASC14, were identified by Genome-Wide Association Study (GWAS) and associated with metastatic disease, amplification of *MYCN* oncogene, and more advanced disease [13,14,15].

The analysis of the other gain regions in the IMR-32 single cells revealed the presence of many genes related to the development and progression of the cancer. For example, in the 1p32.3–1q44 region, notable genes are: *JUN* and *AKT3*, which play a major role in cell proliferation and transformation; *RAPIA* and *RHOC*, implicated in the *RAS* pathway; and *N-RAS*, involved in the signal transduction pathway [16,17,18,19,20]. As reported in Table 1, in the regions 17q21.31–17q25.3 and 15q15.1–15q26.3, there are some genes that have been previously implicated in human cancer, even if until now not in NB.

It is noteworthy that every single cell had a common deletion in the long arm of chromosome 16 (16q22.2–16q24.3), where some notable genes are located, i.e., *ZFHX3* (involved in neuronal differentiation), *WWOX* (involved in apoptosis and downregulated or highly undetected in breast cancer cell lines), and *FXOP1* and *WEDC* (both seem to play a role in prostate cancer) [21,22,23,24].

In just 1 single IMR-32 cell, we found a deletion on chromosome 11 (11p15.2–11p12; 11q14.1–11q23.2; 11q23.2–11q25), which appears to be present in nearly 20–45% of NB patients. This alteration has been related to the development of a more aggressive neuroblastoma with a decreased survival rate [25,26]. By analyzing the genes present in the deleted region, a correlation can be found between the deletion and disease progression. Indeed, in this region, there are 4 genes that deserve to be reported: 2 of these are known to be tumor suppressors, *HTATIP2* (involved in metastasis suppression in several tumors) and *WT1* (whose deletion is associated with nephroblastoma in children); the other 2, *MRE11* and *ATM*, have been reported to be involved in DNA repair mechanisms; thus, their loss of function may lead to defective DNA repair, which in turn, leads to cancer [27,28,29].

Finally, 2 single cells showed a loss of chromosome X, which is peculiar in this study. Among the genes located in the X chromosome that appear to be implicated in cancer (i.e., *VEGFD*, *PRDX4*, *ZBTB33*, *PASD1*), the 1 that mainly could be correlated with NB is *L1CAM*, since it plays a role in axon outgrowth and fasciculation, neuronal migration, and survival, synaptic plasticity, and regeneration after trauma [30]. 

The single cells isolated from the SK-N-BE (2)-C cell line share most of the genetic aberrations identified (Figure 6). The analysis of the chromosomal patterns allowed identifying a main subpopulation (8/14) characterized by the presence of the partial gain of chromosomes 7 and 11, the partial loss in chromosomes 3, 13, 17, 19, and 21, a total loss of the X chromosome, and from the characterizing element, the partial gain of chromosome 1. Moreover, 5/14 cells showed a partial loss in that chromosome (1p32.2–1p21.3) and the partial gain of the chromosomal region 2p25.3–2p21; only 1 of these 5 cells also presented a peculiar gain of chromosome 9.

In the chromosomal region 7q32.1–7q36.3, in addition to *NRF-1* and *BRAF*, involved in several cancers [31], there are another 2 interesting genes, namely *EPHB6* (whose levels have been proposed as prognostic indicators in NB [32]), and *EZH2* (which plays an essential role in the control of the central nervous system by regulating the dopamine D4 receptor [33]). 

*MCAM* and *TMPRSS4*, localized in the chromosomal region 11q13.3–11q25, play a role in invasion, metastasis, migration, and adhesion; of considerable interest, there is also *Fli-1*, which plays an important role in erythropoiesis; in particular, the expression of the *EWS/Fli-1* fusion gene has been shown to be critical for cancer induction in the majority of Ewing’s sarcomas [34,35]. 

The analysis of the altered chromosomal regions found in the SK-N-BE (2)-C cells revealed the presence of many genes related to the development and progression of the cancer, as shown in Table 2.

However, here, we discuss those of greatest interest for NB. For example, *FOXPI* is localized in the chromosomal region 3p26.3–3p14.2.1, a locus often found to be deleted in NB tumors. This locus codes for a set of transcription factors that largely control normal cellular processes, like proliferation and differentiation. The deletion of this locus largely explains the tumor development in NB patients [36]. It has been reported that the *FOXP1* expression level is consistently lower in Stage 4 patients, which corresponds to a poor NB prognostic index. On the contrary, the normal expression of *FOXP1* significantly marks the overall survival rate. *PPARG* and *TGFBR2* (3p26.3–3p14.2), *BRCA2*, and *KLF5* (13q12.11–13q31.1) are linked to many pathological conditions, including cancer; *MLH1* and *BAP1*, whose loss of expression is correlated with microsatellite instability in colorectal cancer and breast cancer, respectively; and finally, *NF1* (17p13.3–17q11.2) related to type 1 neurofibromatosis [37,38].

The analysis of 5/14 cells showed a gain region in chromosome 2: we must remember that SK-N-BE(2)-C is a clonal subline of the SK-N-BE(2) NB cell line. Like the parental cell line, these cells display *MYCN* amplification, which correlates with the gain of the chromosomal region 2p25.3–2p21 (where *MYCN* is located), which is the same alteration we found. The rare forms of familial NB are also featured by *MYCN* overexpression [9].

Moreover, in the 2p35 region, within *BARD1*, several SNPs have been identified and associated with a more aggressive tumor behavior [39,40]. This region is also characterized by the presence of 2 genes, *SOX11* and *ALK*, which may have a role in nervous system development and maintenance; in particular, *ALK* is highly expressed in familial and sporadic NB patients. *ALK* plays an important role in brain development and exerts its effects on specific neurons. It belongs to the tyrosine kinase receptors family with typical transmembrane and extracellular domains. Knocking out *ALK* gene mRNA effectively inhibits cells growth. Constitutive activation of *ALK* is due to translocation [41]. Moreover, the *ID2* gene, a key regulator in the phenotypic transition of neuroblastoma tumor cells, is also present [42].

Only in 1 cell was there found a gain of the chromosomal region 9p24.3–9p23, where *PTPRD* is located, which has been reported to act like a tumor suppressor gene in NB, in addition to other genes, indicated to be related to cancer (i.e., *JAK2*, *RLN2*, *TYRP1*) [43].

Taken together, our results show a high heterogeneity of the CNV status of single cells, although belonging to an immortalized cell line. In fact, despite having analyzed single cells from immortalized cell lines, we found a high inter-cellular heterogeneity, confirming that each cell may potentially accumulate different alterations, which can modulate its behavior, underlining the importance of a precise diagnostic and therapeutic approach for each single patient. 

It is commonly observed that, despite the presence of a given biomarker resulting in being positive after tumor biopsy, patients can be resistant to a given therapy. Our data, obtained from the single-cell analysis, could explain this lack of response to targeted agents, according to the well-known intra-patient heterogeneity. Indeed, rare genomic variations in a single cell could be missed by a bulk analysis of the sample; instead, the single-cell analysis allows identifying alterations present in the less represented clones of the primary tumor. Preclinical studies have already shown the importance of single-cell expression analysis for targeted therapy in breast cancer models [44].

Therefore, the proposed protocol, which aims to evaluate the CNVs on a single cell and then reconstruct its chromosomal patterns, is in line with some data that suggest that gene expression profiles could be more informative in terms of functional status with respect to genetic mutations [45]. Based on our findings, we can speculate that cells with chromosomal alterations, involving the principal genes related to cell proliferation and migration, could mostly contribute to cancer progression. Further studies will be needed to find cell surface antigens able to classify, isolate, and culture different cell types in order to evaluate their contribution to cancer development and/or progression. 

Monitoring the evolution of the cellular heterogeneity of a disease from the early stages could help to identify more aggressive Circulating Tumor Cell (CTC) clones and thus establish a more specific therapeutic approach [46,47,48].

In addition to CTCs, the combination of DEPArray and NGS could be applied also for Formalin-Fixed Paraffin-Embedded (FFPE) tissues as an additional tool for cancer genetic diagnostic purposes [49].

In summary, these data highlight the substantial intra-tumor heterogeneity that occurs at the single-cell level and support the proposed protocol for the analysis of CNVs and the determination of chromosomal patterns at the single-cell level, as a diagnostic and therapeutic strategy for precision medicine.

## 4. Materials and Methods

The protocol, developed on 2 NB cell lines, SK-N-BE (2)-C and IMR-32, includes: (a) the identification and separation of each single cell in a single tube; (b) the preliminary Whole Genome Amplification (WGA) step; (c) the NGS for the detection of the CNVs; and (d) the software for the analysis of the chromosomal patterns.

### 4.1. Cell Lines

The 2 NB cell lines, SK-N-BE (2)-C and IMR-32, used in this study were kindly provided by the cell culture facility of CEINGE-Biotecnologie Avanzate s.c.a.r.l.; the cell lines were cultured at 37 °C with 5% CO_2_ in a humidified atmosphere. NB cell lines were grown in Minimal Essential Eagle Medium (MEM; Sigma, St. Louis, MO, USA) with 10% heat-inactivated FBS (Sigma, St. Louis, MO, USA), 1 mmol/L l-glutamine, penicillin (100 U/mL), and streptomycin (100 mg/mL; Thermo Fisher Scientific, Carlsbad, CA, USA). The cell lines used for all the experimental procedures described herein were tested as mycoplasma free. Experiments were performed on early passage cells. 

### 4.2. Isolation of Intact Single Cells by DEPArray^TM^

The DEPArray™ System is an automated instrument able to identify, sort, and recover individual rare cells, after a preliminary cell immunofluorescence staining. Once collected from the cell culture plate, 500,000 cells for each cell line were suspended in the Running Buffer (RB: PBS with BSA 0.5% and EDTA 2 mM), fixed in 2% PFA at Room Temperature (RT) for 20′, suspended in blocking solution with 3% BSA at RT for 10′, and processed for the immunofluorescence staining. The cells were firstly filtered (by a 30-micrometer filter) in the RB and then incubated with 100 μL of primary antibody (mouse anti-human GD2; BD Biosciences, San Jose, CA, USA) at 4 °C for 60′. GD2 has been used extensively as a target in mAb therapy and has been the primary target of antibody recognition in NB [50]. The cells were then incubated at 4 °C for 60′ with secondary antibody (goat anti-mouse APC; Abcam, Cambridge, UK), suspended in a permeabilizing solution with 100 μL of triton 0.2% for 5′, and stained with 100 μL of Hoechst solution for 5′ at RT. After incubation, cells were washed twice by adding 1 ml of RB and centrifuged at 2000 rpm for 5′. An aliquot of each sample (~1 mL) was transferred into a clean 1.5-mL tube, filled, washed twice with 1 mL of SB115 buffer (Silicon Biosystems, Bologna, Italy) at RT, and centrifuged at 1000× *g* for 5′. Between different steps, when not detailed above, cells were washed thrice with 1 mL of PBS and centrifuged at 2000 rpm for 5′.

For the sorting process, 5000 cells in 13 mL were loaded with 380 mL of the manipulation buffer (SB115, Silicon Biosystem, Bologna, Italy) into an A300K cartridge (Silicon Biosystem, Bologna, Italy). This single-use, microfluidic cartridge contains an array of individually-controllable electrodes, each with embedded sensors. This circuitry enables the creation of Dielectrophoretic (DEP) cages around the cells. Individual cells of interest are gently moved to specific locations on the cartridge or into the holding chamber for their isolation and recovery. The cartridge was then scanned by an automated fluorescence microscope; this optic system provides a 10× magnification (0.64 micron/pixel) and a 20× magnification (0.32 micron/pixel) resolution. The CellBrowser software (Silicon Biosystem, Bologna, Italy) allows cell selection based on multiple parameters from fluorescence and bright field images. The protocol chosen was fixed low-density cells, and the chip-scan setting included DAPI, Brithfield, and APC. Firstly, cells able to move were grouped, and then, based on APC, fluorescent cells were isolated as GD2 positive. High-quality, image-based selection allows the identification and isolation of the cells of interest. Taking into account the SB115 starting volume, the DEPArray allows isolating a maximum of 35 single cells. Each cell was individually collected, washed twice in PBS, and stored at −20 °C until the WGA. 

### 2.3. Whole Genome Amplification

Cell lysis and genome amplification were performed using the SurePlex^TM^ DNA Amplification System (Illumina, San Diego, CA, USA), following the manufacturer’s instructions. A negative no template control (2.5 μL of PBS) and a SurePlex-positive control (15 pg of genomic DNA) were used for each reaction. In brief, each single cell underwent lyses and DNA extraction, SurePlex pre-amplification, and finally, the SurePlex amplification step. To determine the success of the amplification, 5 μL of each amplified sample plus 5 μL gel loading buffer (2×) were loaded on a 1.5% agarose 1× TBE gel. WGA products were quantified using the Qubit dsDNA High Sensitivity Assay kit (Thermo Fisher Scientific, Carlsbad, CA, USA). 

### 4.4. Next Generation Sequencing

WGA samples were analyzed by NGS using the VeriSeq PGS Kit (Illumina, San Diego, CA, USA), a system specifically designed for single-cell analysis and able to provide a comprehensive and accurate screening of all 24 human chromosomes in approximately 12 h. Tagmentation, sample barcoding, and libraries’ preparation were all performed using the manufacturer’s protocol. Then, the products were purified by using a size selection and normalized to equalize the quantity of each sample. The final products were pooled, denatured, and sequenced using the MiSeq Reagent Kit v3, PGS (Illumina, San Diego, CA, USA) on a MiSeq System.

### 4.5. Data Analysis

NGS results were analyzed using the BlueFuse Multi Software V4.4 (Illumina, San Diego, CA, USA), a complete solution for analyzing and reporting the VeriSeq results by enabling a full understanding of the status of each chromosome and the results’ confirmation. Sophisticated algorithms calculate and call the status for each chromosome, as either normal or abnormal, and include an estimate of the confidence in the call based on the assay noise or on any underlying ambiguity. In particular, for sequencing data, the number of sequences is proportional to the copy number, so a greater or lower number of reads will correspond to the gain or loss of chromosomal regions.

Once CNVs were detected in each analyzed single cell, we verified for each altered chromosomal region which genes were comprised in them. The genes were referenced from the Atlas Genetics Oncology.org (http://atlasgeneticsoncology.org, accessed on September 2018), an open access website that contains all the information about the genes that have been related to cancer. Genes were selected based on their position in chromosomes that correlated with our NGS findings, as well as on their direct correlation with tumor progression and tumor development in various cancers, apart from NB.

## 5. Conclusions

Our data show that the combined use of DEPArray^TM^ technology with high-coverage NGS provides a good method to identify and explore CNVs, from which it is possible to screen the chromosomal patterns in cancer cells, and it is a well-established approach to examine tumor genetic heterogeneity. Furthermore, the chromosomal pattern evaluation of the collected single cells may be useful to highlight the driving mutations responsible for disease progression and therapy response. 

These preliminary data encourage the application of this protocol also in other types of cancers and support the idea that the identification of chromosomal patterns, rather than individual biomarkers, could demonstrate the value of liquid biopsy as a diagnostic and prognostic tool.

## Figures and Tables

**Table 1:** The table shows the gain or loss chromosomal regions identified in the 19 IMR-32 single cells analyzed and the main genes implicated in the mechanisms of cancer regulation, according to recent literature.

		Gene		
Single Cell	Chr. Alteration	Name	Position	Features and Implications with Cancer
19/19	1p32-3–1q44 (194 Mb)	*JUN*	1p32.1	Cell proliferation and transformation
		*RAPIA*	1p13.2	Activator of Raf gene in the MAP kinase pathway
		*RHOC*	1p13.2	Believed to have a similar function as Ras
		*NRAS*	1p31.2	Signal transduction pathways
		*AKT3*	1q43–q44	Cell proliferation, survival, and tumorigenesis
	6	*FOXQ1*	6p25.3	Increased migration and proliferation
		*SOX4*	6p22.3	Increased survival in medulloblastoma
		*AGER*	6p21.32	Promoter of human glioblastoma cell growth and migration
		*SRSF3*	6p21.31	Cell cycle progression control
		*NCR2*	6p21.1	Cell cycle regulation
		*HACE1*	6q16.3	Tumor suppressor
		*LATS1*	6q24-25.1	Overexpression reduces cell proliferation, migration. and invasion
	17q21.1–17q25.3 (42 Mb)	*SLC4A1*	16q22.2	Overexpression leads to tumor progression
		*NMT1*	16q23.1	Upregulated due to gene amplification
		*FMNL1*	16q24.1	Overexpression leads to cell growth
		*NGFR*	16q24.1	Acts as a tumor marker for neural crest cells
	16q21–16q24.2 (26 Mb)	*ZFHX3*	17q21.31	Neuronal differentiation
		*WWOX*	17q21.31	Possible involvement in apoptosis
		*FXOP1*	17q21.31	Cell cycle progression, invasion, and metastasis
		*WEDC1*	17q21.33	Functions like the tumor suppressor gene
15/19	15	*PLCB2*	15q15.1	Overexpressed in cancer tissues
		*TYRO3*	15q15.1	Highly expressed in certain cancers
4/19	15q15.1–15q26.3 (60 Mb)	*RASGRF1*	15q25.1	Overexpression in the MAPK cascade in neuronal cells
		*PCSK6*	15q26.3	High expression in breast cancer and prostate cancer
1/19	11p15.2–11p12 (42 Mb); 11q14.1–11q23.2 (32 Mb); 11q23.2–11q25 (21 Mb)	*HTATIP2*	11p15.1	Suppression of metastasis in various tumors
		*WT1*	11p13	Tumor suppressor
		*MRE11*	11q21	DNA repair mechanism
		*ATM*	11q22.3	DNA repair mechanism
1/19	13	*See Table 2*		
2/19	X	*VEGFD*	Xp22.2	Angiogenesis, lymphangiogenesis, and metastasis
		*PRDX4*	Xp22.11	Facilitates protein folding
		*ZBTB33*	Xq24	Both an activator and repressor of transcription
		*PASD1*	Xq28	Transcription factor
		*L1CAM*	Xq28	Axon outgrowth and neuronal migration
		*VEGFD*	Xp22.2	Angiogenesis, lymphangiogenesis, and metastasis
Legend				
	Partial gain			
	Total gain			
	Partial loss			
	Total loss			

**Table 2:** The table shows the gain or loss chromosomal regions identified in the 14 SK-N-BE (2)-C single cells analyzed and the main genes implicated in the mechanisms of cancer regulation, according to recent literature.

		Gene		
Single Cell	Chromosomal Alteration	Name	Position	Features and Implications with Cancer
14/14	7q32.1–7q36.3 (31 Mb)	*NRF1*	7q32.3	Cell cycle regulation
		*BRAF*	7q34	Belongs to the RAS/RAF/MEK/ERK/MAPK pathway
		*EPHB6*	7q34	Prognostic indicators in neuroblastoma
		*EZH2*	7q36.1	Role in the control of the central nervous system
		*XRCC2*	7q36.1	Involved in homologous recombination
	11q13.3–11q25 (65 Mb)	*MRE11*	11q21	DNA repair mechanism
		*ATM*	11q22.3	DNA repair mechanism
		*MCAM*	11q23.3	Cell adhesion molecules
		*FLI1*	11q24.3	Role in erythropoiesis
		*TMPRSS4*	11q23.3	Role in invasion, metastasis, migration, and adhesion
	3p26.3–3p14.2 (61 Mb)	*PPARG*	3p25.2	Anti-inflammatory role
		*TGFBR2*	3p24.1	Loss of expression is linked with cancer
		*MLH1*	3p22.2	Recruitment of proteins for excision and repair
		*BAP1*	3p21.1	Enhances BRCA1-mediated inhibition
	13q12.11–13q31.1 (66 Mb)	*LATS2*	13q12.11	Overexpression inhibits tumor formation
		*PDX1*	13q12.2	Overexpression is correlated with metastasis
		*BRCA2*	13q13.1	Maintenance of genomic integrity
		*RB1*	13q14.2	Cell cycle regulation and differentiation
		*KLF5*	13q22.1	Cell cycle, cell proliferation, and apoptosis
		*LATS2*	13q12.11	Overexpression inhibits tumor formation
	17p13.3–17q11.2 (30 Mb)	*FAM57A*	17p13.3	Amino acid transport and glutathione metabolism
		*CRK*	17p13.3	Overexpressed in various human cancers
		*MAP2K4*	17p12	Response to cellular stress
		*NF1*	17q11.2	Loss of function leads to neurofibromatosis type 1
		*KSR1*	17q11.2	Might be involved in Ras-mediated oncogenesis
	19q12–19q13.43 (28 Mb)	*PDCD5*	19q13.11	Promotes apoptosis; underexpressed
		*FXYD3*	19q13.12	Downregulated in various cancers
		*PAF1*	19q13.2	Overexpression results in enhanced growth rates
		*BAX*	19q13.33	Proapoptotic function
		*ATF5*	19q13.33	Proliferation and differentiation of neural cells
	21q22.2–21q22.3 (6 Mb)	*ERG*	21q22.2	Regulator of mitogenic signal transduction pathways
		*ETS2*	21q22.3	Positive or negative regulator of gene expression
		*TMPRSS2*	21q22.3	Involved in prostate cancer
		*CSTB*	21q22.3	Related to a favorable prognosis for cancer patients
		*PTTG1IP*	21q22.3	Overexpressed in thyroid tumors
	X	*See Table 1*		
8/14	1p21.3–1q44 (151 Mb)	*ABL2*	1q25.2	Involved in acute non-lymphocytic leukemia
		*TP53BP2*	1q43–44	Apoptosis, cell cycle, tumor suppression, and cell polarity
5/14	1p32.2–1p21.3 (44 Mb)	*JUN*	1p32.1	Cell proliferation and transformation
		*JAK1*	1p31.3	Signaling by the majority of cytokines
		*GADD45A*	1p31.3	Maintenance of genome integrity
		*NRAS*	1p31.2	Signal transduction pathways
6/14	2p25.3–2p21 (44 Mb)	*SOX11*	2p25.2	Development in the nervous system of the human fetus
		*ID2*	2p25.1	Phenotypic transition of neuroblastoma tumor cells
		*N-Myc*	2p24.3	Expressed in several tumors
		*ALK*	2p23.2	Development and maintenance of the nervous system
		*EPCAM*	2p21	Oncogenic signaling molecule
1/14	9p24.3–9p23 (13 Mb)	*JAK2*	9p24.1	Associated with cytokine receptors
		*RLN2*	9p24.1	Induced by a variety of factors in different tissues
		*PTPRD*	9p24.1	Tumor suppressor gene in neuroblastoma
		*TYRP1*	9p23	Correlated with distant metastasis-free survival
Legend				
	Partial gain			
	Total gain			
	Partial loss			
	Total loss			

## Abbreviations

IMR-32	Neuroblastoma cell line
SK-N-BE(2)-C	Neuroblastoma cell line
CNVs	Copy Number Variants
GD2	Disialoganglioside
DEPArray	DiElectrophoresis Array
WGA	Whole Genome Amplification
NB	Neuroblastoma
NCCs	Neural Crest Cells
ALK	Anaplastic Lymphoma Kinase
SNP	Single-Nucleotide Polymorphisms
GWAS	Genome-Wide Association Study
FOXPI	Fork Head Box P1
TP53	Tumor Protein 53
AKT or PKB	Protein Kinase B
NGS	Next-Generation Sequencing
BSA	Bovine Serum Albumin
NTC	No Template Control
EDTA	EthyleneDiamineTetraacetic Acid
DAPI	4′,6-Diamidino-2-Phenylindole
APC	AlloPhycoCyanin
RB	Running Buffer
